# Age Estimation in Sportspersons From the Epiphyseal Fusion Around Wrist, Elbow, and Pelvic Joints

**DOI:** 10.7759/cureus.33282

**Published:** 2023-01-02

**Authors:** Abhijit Hosmani, Harish Pathak, Harshwardhan Khartade, Devendra Jadav, Rutwik Shedge, Mohan Pawar, Vikas Meshram

**Affiliations:** 1 Forensic Medicine and Toxicology, Aundh Chest Hospital, Pune, IND; 2 Forensic Medicine and Toxicology, Seth Gordhandas Sunderdas Medical College and King Edward Memorial Hospital, Mumbai, IND; 3 Forensic Medicine and Toxicology, Shyam Shah Medical College, Rewa, IND; 4 Forensic Medicine and Toxicology, All India Institute of Medical Sciences, Jodhpur, Jodhpur, IND; 5 Forensic Anthropology, School of Forensic Sciences, National Forensic Sciences University, Tripura, IND; 6 Forensic Medicine and Toxicology, Dr. Balasaheb Vikhe Patil Rural Medical College, Loni, IND

**Keywords:** age fraud, sportsperson, bone age estimation, anthropology, forensic identification

## Abstract

Background

Age estimation of an individual is an integral part of medicolegal work. Out of many scenarios for which age estimation is performed, competitive sports is the one emerging field where experts are consulted for providing accurate age of the athlete. Owing to the chances of deliberately increasing (padding) or decreasing (shaving) the age of the athlete for his own advantage, accurate age estimation is crucial. The Sports Authority of India (SAI) mandates age verification from experts prior to participation in sports events in various age group categories. One of the widely used methods of age estimation in athletes is the radiological examination of the ossification centers of bones.

Methodology

The study was performed on 134 athletes (72 males and 62 females) with an age range of 12-18 years old with due permission from the Sports Authority of India (SAI) for this study. These participants compete at state, national, and international levels in squash, handball, swimming, cricket, and judo in under-14, under-16, and under-19 age categories. X-rays of the wrists, elbows, and pelvis were analyzed using the Schmeling five-stage method for the fusion of ossification centers.

Results

A greater degree of correlation between the fusion stages of all regions of interest and chronological age was observed in males than in females. The highest correlation in both sexes is observed between the fusion score of the head of the radius and the age (R = 0.814 for males and R = 0.647 for females). The lowest correlation for both males and females is seen between the fusion score of the lateral epicondyle of the humerus and age (R = 0.754 for males and R = 0.441 for females). Multiple linear regression models showed a standard error of estimate (SEE) of 1.093 years for the elbow joint, 1.147 years for the wrist joint, 1.039 years for the pelvis joint, and 1.030 years for all three joints.

Conclusion

Regression models generated for estimating the age of sportspersons from the ossification centers of the elbow, wrist, and pelvis in the present study can be applied for the age estimation of individuals aged between 12 and 18 years. Future population-specific studies on the age estimation of sportspersons with greater sample sizes are necessary to validate the findings of the present study.

## Introduction

Age estimation is one of the essential factors in establishing the exact identity of a living individual. Apart from identification, age estimation is required for civil purposes such as employment, consent for marriage, attainment of majority, participation in competitive sports, and immigration and for criminal scenarios such as cases of rape and kidnapping and for criminal responsibility, prostitution, and judicial punishment [1].

To uphold the principle of sporting fairness and safeguard athletes’ health, age estimation is of utmost importance in competitive sports as well [2]. Competitive sports are divided into different categories based on age groups to ensure fair play among similar age group individuals. Age classification varies from sport to sport and may range from under-14s to under-21s. Age estimation is often done on participants of such competitive sports events to ensure that older, more athletic athletes do not falsify their age to compete against younger, comparatively immature athletes [2].

Age estimation in sportspersons becomes more important in developing countries where birth records are often not well maintained. In recent times, magnetic resonance imaging (MRI) is emerging as a method of choice for age estimation in individuals participating in competitive sports, especially in developed countries, where such resources are available [3-5]. On the other hand, in developing countries such as India, age estimation by observing the appearance and fusion of ossification centers on conventional radiography is still considered one of the most accurate and reliable methods for age estimation among sportspersons [6].

This study aims to evaluate the fusion of ossification centers of the elbow, wrist, and pelvis in sportspersons of the Mumbai region of Maharashtra, India, and formulate regression models that can be used for the estimation of age in sports personnel.

## Materials and methods

The study was conducted in the Department of Forensic Medicine and Toxicology of a tertiary care center after securing ethical clearance from the institutional ethics committee. Permission was taken from the Sports Authority of India (SAI) for this study. The total number of participants studied was 134 (72 males and 62 females), with an age range of 12-18 years. These participants were athletes competing at state, national, and international levels in squash, handball, swimming, cricket, and judo in under-14, under-16, and under-19 age categories. The date of birth of each participant was confirmed by verifying their official birth proof. The participants were examined to rule out any abnormalities and disabilities involving the upper limbs and the pelvis. X-rays of the left wrist and pelvis in anteroposterior view and X-rays of the left elbow in anteroposterior and lateral views were taken at the Department of Radiology after taking written informed consent from the parents and legal guardians of sportspersons. X-rays were analyzed by two investigators separately after blinding.

The study participants included sportspersons in the age group of 12-18 years. Hence, the following ossification centers were observed. For the elbow joint, the ossification centers of the trochlea, medial epicondyle of the humerus, lateral epicondyle of the humerus, head of the radius, and olecranon were analyzed. For the wrist joint, the ossification centers of the distal radial epiphysis, distal ulnar epiphysis, and base of the first metacarpal were analyzed. For the pelvis joint, the ossification centers of the head of the femur, greater trochanter, and lesser trochanter were analyzed. All ossification centers were graded according to the Schmeling classification system [7] as stated in Table 1.

**Table 1:** Schmeling five-stage classification system for ossification

Stage	Description
Stage 1	No ossification of the ossification center
Stage 2	Presence of ossified ossification center but absence of ossification of the epiphyseal cartilage
Stage 3	Partial ossification of the epiphyseal cartilage
Stage 4	Complete ossification of the epiphyseal cartilage with the presence of epiphyseal scar
Stage 5	Absence of epiphyseal scar

Statistical analysis

Each stage was assigned an ordinal value for the purpose of analysis: stage 1 was graded as 1, stage 2 as 2, and so on. This ordinal value was considered the fusion score. Descriptive statistics for the entire data were calculated. Spearman’s correlation was calculated between age and the fusion score for each ossification center for all participants. Simple linear regression models were generated for all ossification centers to estimate an age for both sexes. Multiple regression models were generated using the ossification centers of the elbow, wrist, and pelvis separately and together for both sexes. The data of the study were analyzed using the Statistical Package for the Social Sciences (SPSS) version 25 (IBM SPSS Statistics, Armonk, NY, USA).

## Results

The participants’ age ranged from 12 to 18 years, with 72 males showing a mean ± standard deviation (SD) age of 14.80 ± 1.89 years and 62 females showing a mean ± SD age of 14.13 ± 1.65 years. Descriptive statistics for different stages of epiphyseal fusion for each of the ossification centers for males and females showed that the mean fusion scores for the ossification centers of the elbow joint are the highest for both sexes, followed by the pelvis and then the wrist. This indicates that the bones of the elbow joint ossify first, followed by the pelvis and then the wrist.

**Figure 1:**
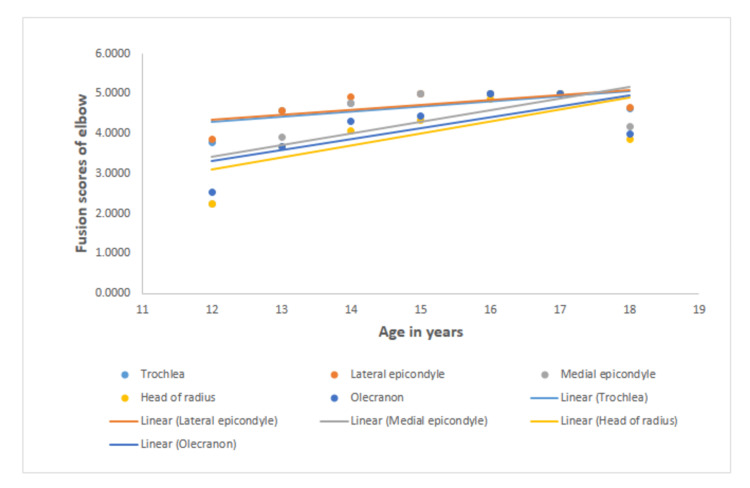
Graphical representations of the fusion scores of the ossification centers of the elbow with chronological age

**Figure 2:**
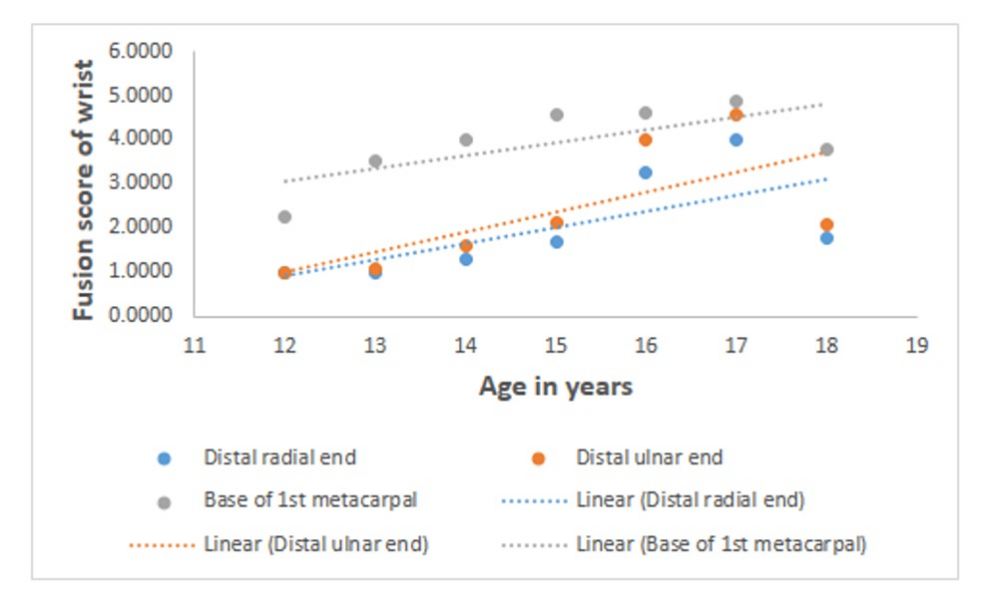
Graphical representations of the fusion scores of the ossification centers of the wrist with chronological age

**Figure 3:**
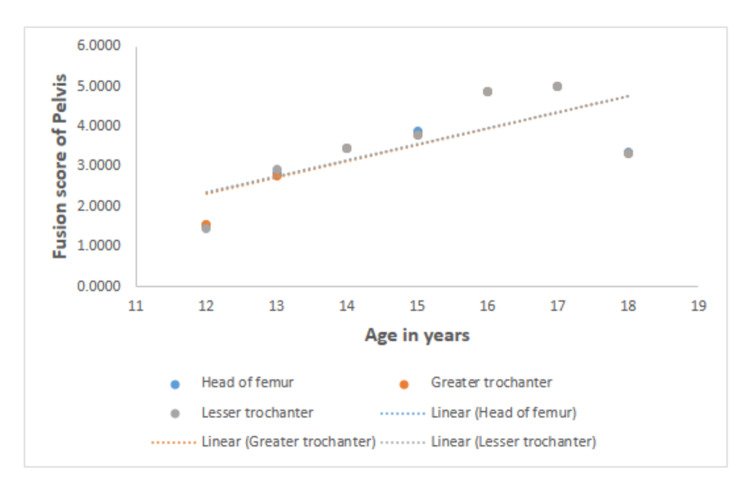
Graphical representations of the fusion scores of the ossification centers of the pelvis with chronological age

Table 2 shows the correlation between age and fusion scores for the ossification centers of the elbow, wrist, and pelvis for both sexes. A greater degree of correlation between the fusion stages of all regions of interest and chronological age was observed in males than in females. The highest correlation in both sexes is observed between the fusion score of the head of the radius and age (0.814 for males and 0.647 for females). The lowest correlation for both males and females is seen between the fusion score of the lateral epicondyle of the humerus and age (0.754 for males and 0.441 for females). In the wrist joint, the highest correlation was seen between the base of the first metacarpal for males (0.762) and between the distal ulnar end and age for females (0.819). In the wrist joint, the least correlation was seen between the fusion score of the distal ulnar end and age for males (0.632) and between the fusion score of the base of the first metacarpal and age for females (0.684). In the pelvis, the highest correlation was seen between the fusion score of the head of the femur and age for both males (0.838) and females (0.76). In the pelvis, the lowest correlation was seen between the fusion score of the greater trochanter and age for males (0.816) and between the fusion score of the lesser trochanter and age for females (0.737). All values are statistically significant (p < 0.01). The graphical representation of the fusion scores of the elbow, wrist, and pelvis with chronological age is depicted in Figures 1-3.

**Table 2:** Correlation between age and the ossification scores of the regions of the elbow, wrist, and pelvis for males and females

Region of interest	R for male	R for female	p-value
Trochlea	0.757	0.499	<0.01
Lateral epicondyle of the humerus	0.754	0.441	<0.01
Medial epicondyle of the humerus	0.764	0.599	<0.01
Head of the radius	0.814	0.647	<0.01
Olecranon	0.810	0.618	<0.01
Distal radial epiphysis	0.644	0.778	<0.01
Distal ulnar epiphysis	0.632	0.819	<0.01
Base of the first metacarpal	0.762	0.684	<0.01
Head of the femur	0.838	0.760	<0.01
Greater trochanter	0.816	0.738	<0.01
Lesser trochanter	0.817	0.737	<0.01

Table 3 shows the simple linear regression equations using the fusion scores of all ossification centers examined in the study to estimate the age of males and females.

**Table 3:** Simple linear regression models using the fusion scores of all ossification centers for both sexes T: trochlea, LE: lateral epicondyle of the humerus, ME: medial epicondyle of the humerus, RH: head of the radius, O: olecranon, DR: distal radial epiphysis, DU: distal ulnar epiphysis, MTC: base of the first metacarpal, HF: head of the femur, GT: greater trochanter, LT: lesser trochanter, SEE: standard error of estimate

Ossification center	Male		Female	
	Model	SEE (years)	Model	SEE (years)
T	0.879 (T) + 11.471	1.243	1.036 (T) + 9.351	1.446
LE	0.840 (LE) + 11.620	1.252	0.859 (LE) + 10.124	1.497
ME	0.752 (ME) + 12.346	1.229	0.653 (ME) + 11.411	1.336
RH	0.839 (RH) + 12.181	1.105	0.784 (RH) + 11.106	1.272
O	0.823 (O) + 12.178	1.117	0.769 (O) + 11.055	1.311
DR	0.943 (DR) + 13.116	1.457	1.025 (DR) + 12.294	1.047
DU	0.78 (DU) + 13.286	1.476	0.910 (DU) + 12.221	0.956
MTC	0.817 (MTC) + 12.148	1.233	0.9 (MTC) + 10.717	1.217
HF	0.933 (HF) + 11.98	1.039	0.824 (HF) + 11.365	1.083
GT	0.893 (GT) + 12.162	1.101	0.775 (GT) + 11.555	1.125
LT	0.865 (LT) + 12.258	1.098	0.772 (LT) + 11.551	1.128

Table 4 and Table 5 show the multiple linear regressions using the fusion scores of all ossification centers examined in the study to estimate the age of males and females, respectively. 

**Table 4:** Multiple linear regression model for the ossification centers for males OC: ossification center, T: trochlea, LE: lateral epicondyle of the humerus, ME: medial epicondyle of the humerus, HR: head of the radius, O: olecranon, DR: distal radial epiphysis, DU: distal ulnar epiphysis, MTC: base of the first metacarpal, HF: head of the femur, GT: greater trochanter, LT: lesser trochanter, SEE: standard error of estimate

Region	R	R^2^	Model	SEE (years)
Elbow	0.830	0.690	0.269 (T) + 0.068 (LE) – 0.101 (ME) + 0.625 (HR) + 0.066 (O) + 11.693	1.093
Wrist	0.804	0.647	0.387 (DR) + 0.063 (DU) + 0.625 (MTC) + 11.958	1.147
Pelvis	0.843	0.71	1.606 (HF) – 0.636 (GT) – 0.033 (LT) + 11.92	1.039
Elbow + wrist + pelvis	0.866	0.749	0.433 (LE) – 0.154 (T) – 0.11 (ME) + 0.072 (HR) + 0.249 (O) + 0.390 (DR) -0.005 (DU) -0.011 (MTC) +1.047 (HF) – 0.342 (GT) – 0.399 (LT) + 11.454	1.030

**Table 5:** Multiple linear regression model for the ossification centers for females OC: ossification center, T: trochlea, LE: lateral epicondyle of the humerus, ME: medial epicondyle of the humerus, HR: head of the radius, O: olecranon, DR: distal radial epiphysis, DU: distal ulnar epiphysis, MTC: base of the first metacarpal, HF: head of the femur, GT: greater trochanter, LT: lesser trochanter, SEE: standard error of estimate

Region	R	R^2^	Model	SEE (years)
Elbow	0.660	0.435	0.237 (T) -0.523 (LE) + 0.157 (ME) + 0.633 (HR) + 0.146 (O) + 11.802	1.298
Wrist	0.866	0.75	0.216 (DR) + 0.527 (DU) + 0.45 (MTC) + 10.931	0.847
Pelvis	0.866	0.75	0.216 (DR) + 0.527 (DU) + 0.45 (MTC) + 10.931	0.847
Elbow + wrist + pelvis	0.894	0.799	0.119 (LE) – 0.159 (T) + 0.294 (ME) – 0.427 (HR) + 0.036 (O) + 0.214 (DR) + 0.474 (DU) + 0.367 (MTC) + 1.06 (HF) – 0.075 (GT) – 0.71 (LT) + 10.883	0.818

Table 6 depicts the ages of fusion for all ossification centers at stages 4 and 5 for both sexes. The mean age of fusion is the least in the ossification centers of the elbow, followed by the mean age in the ossification centers of the wrist and then the ossification centers of the pelvis for both sexes.

**Table 6:** Mean age of fusion for all ossification centers for both sexes

Ossification center	Mean age in years at fusion stage 4		Mean age in years at fusion stage 5	
	Male	Female	Male	Female
Trochlea	14.18	12.88	16.07	14.62
Lateral epicondyle	13.89	12.5	16.04	14.54
Medial epicondyle	15.33	13.33	16.1	14.75
Head of the radius	15.44	13.72	16.42	15.41
Olecranon	15.42	13.58	16.3	15.25
Distal radial end	16.85	16.71	18	16.5
Distal ulnar end	16.83	16.25	16.9	16.57
Base of the first metacarpal	15.18	13.9	16.38	15.78
Head of the femur	15.35	14.04	16.75	16.05
Greater trochanter	15.38	14.0	16.75	16.05
Lesser trochanter	15.2	14	16.76	16.05

## Discussion

Age estimation is crucial in athletes participating in competitive sports events. These events divide athletes based on their age to ensure competition between similarly aged individuals. Such sports events are becoming more and more professional and commercial as time progresses. Highly talented individuals get scouted and get signed by professional clubs or teams through these events. This creates a lot of strain on the athletes to succeed and may pressure them enough to fabricate their age to ensure success. Sometimes, the age of the sportsperson is deliberately decreased (shaving) to enable participation in the lower age category or increased (padding) to enable participation in a higher category [8]. Padding is done in events where there is a lower limit for participation, such as the Olympics, where the minimum age is 14 years. This is often achieved by doctoring the documents of proof of birth. Hence, in most parts of the world, it is mandatory to verify the age by radiological examination with documentary proof prior to participation. In India, the Ministry of Youth Affairs and Sports and the Sports Authority of India have made verification of the age by a medical expert mandatory prior to participation in events [8]. In India, various frontier sports bodies, including the Board of Control for Cricket in India (BCCI) and the All India Football Federation (AIFF) have approved the assessment of X-rays of the wrist and hand to estimate age in Indian sportspersons [6]. While the AIFF relies solely on the Tanner-Whitehouse 3 (TW3) method that includes wrist and hand X-rays, the Sports Authority of India (SAI) specifically advises X-rays of the shoulder, elbow, hand and wrist, and pelvis with hip joint for age estimation practices along with dental examination and general physical examination and also reserves body development index method as an optional method for individuals below the age of 18 years.

In the present study, the elbow joint, wrist joint, and pelvis of 134 athletes were radiographed to study their ossification. The stages of fusion were scored using the Schmeling five-stage classification system, and simple and multiple linear regression models were generated for the estimation of age using those scores. It was observed that the ossification centers of the elbow joint fused the earliest, followed by the ossification centers of the pelvis, and then the ossification centers of the wrist.

The Schmeling method is a five-stage method for age estimation and is widely approved to study epiphyseal fusion of long bones, but most of the research is limited to the clavicle [9,10]. Apart from the clavicle, only two studies have been performed using the Schmeling five-stage method of ossification, also only on the wrist joint [11,12]. To the best of our knowledge, the Schmeling five-stage method has not been used to estimate the age of sportspersons as well.

Schmidt et al. (2008) examined the epiphyseal fusion of wrist bones in 265 German males and 164 females, ages ranging from 10 to 19 years, using the Schmeling method [11]. Similarly, Baumann et al. (2009) carried out a similar study to examine the epiphyseal fusion of the wrist bones of 554 males and 288 females, ages ranging from 10 to 31 years [12]. The comparison of the mean age of fusion for stage 4 and stage 5 in all three studies are shown in Table 7.

**Table 7:** Mean age with SD of fusion for stages 4 and 5 in the distal end of the radius and ulna in the literature SD: standard deviation

Study with age range of participants	Region of interest	Mean age of fusion with SD in years	Stage 4		Stage 5	
			Male	Female	Male	Female
Baumann et al. [12] (10 to 31 years)	Distal radius	Mean age	14.5 to 31	12.9 to 30.8	18.7 to 30.7	16.2 to 30.8
		SD	22 ± 4.1	20.4 ± 4.3	24.7 ± 3.7	23.4 ± 4.3
	Distal ulna	Mean age	14.5 to 29	13.6 to 29.9	15.2 to 0.31	13.9 to 30.8
		SD	17 ± 2.3	16.8 ± 3.5	23.1 ± 3.7	21.3 ± 4.2
Schmidt et al. [11] (10 to 19 years)	Distal radius	Mean age	14.5 to 18.9	12.9 to 19	18.7 to 19	16.2 to 19
		SD	17.2 ± 1.3	16.5 ± 1.1	18.8 ± 0.2	17.8 ± 1.2
	Distal ulna	Mean age	14.5 to 18.8	13.6 to 17	15.2 to 19	13.9 to 19
		SD	16.6 ± 1.3	15.6 ± 1.1	18 ± 0.9	17 ± 1.2
Present study (12 to 18 years)	Distal radius	Mean age	15 to 18	16 to 17	17 to 18	16 to 17
		SD	16.8 ± 0.86	16.7 ± 0.48	16.5 ± 0.8	16.5 ± 0.71
	Distal ulna	Mean age	16 to 18	15 to 17	15 to 18	16 to 17
		SD	16.8 ± 0.75	16.2 ± 0.71	16.9 ± 0.99	16.5 ± 0.53

The mean age for the fusion of Schmidt et al. [11] and the present study was similar, as the age range included in these studies was identical. On the other hand, the mean age for fusion was much higher in the study of Baumann et al. [12], as the age range included up to 30 years. A literature search did not reveal any study that has used the Schmeling staging of epiphyseal fusion for age estimation from the elbow and pelvis joint.

We also compared the age estimation studies for non-sports personnel in the Indian population with our study. Hassan et al. (2016) studied the epiphyseal fusion of the distal radial and ulnar epiphyses in the Indian population [13]. It was observed in the study that 40% showed complete ossification for the radial epiphysis and 50% showed complete ossification for the ulnar epiphysis in the age group of 16-17 years for males. In the present study, 41% showed complete ossification for the radial epiphysis, and 50% showed complete ossification for the ulnar epiphysis in the age group of 16-17 years for males. In the same age group of females, the study of Hassan et al. reported complete ossification in 60% for the radial epiphysis and 70% for the ulnar epiphysis [13]. In the present study, for the females of the age group 16-17 years, 60% showed complete ossification for the radial epiphysis, and 93% showed complete ossification for the ulnar epiphysis.

It is a well-known fact that epiphyseal fusion occurs more quickly in females than in males of all ages [14]. These conclusions are also supported by the current research in which the mean age for fusion was less in females than in males in all ossification centers studied (Table 5). The findings of this study support those previously published by other authors and indicate that some remnants of the epiphyseal scar may persist until at least the fifth decade of life [15]. The same has been observed in the present study as the mean age of fusion at stage 5 is higher than the mean age of fusion at stage 4, with some being up to 18 years of age (Table 6).

Limitations

The project was a part of the MD thesis and hence was time-bound and also involved the specific category of sportspersons. So, the sample size was restricted to 134 only. The study participants in this study belonged to the age group of 12-18 years. Hence, the appearance of carpal bones for age estimation were not considered in this research. Also, this study has used the Schmeling five-stage method of ossification, which could not be applied to the iliac crest, ischial tuberosity, and triradiate cartilage.

## Conclusions

Regression models generated for estimating the age from the ossification centers of the elbow, wrist, and pelvis can be applicable to sportspersons. Future population-specific studies on the age estimation of sportspersons with greater sample sizes are necessary to validate the findings of the present study. It can help ensure better objectivity in a relatively subjective field of age estimation and help in the accurate estimation of age in the Indian scenario. This will further help in detecting cases of age fabrication and ensuring fair opportunities for every sportsperson.
